# Evaluating the performances of PI controller (2DOF) under linear and nonlinear operations of DFIG-based WECS: A simulation study

**DOI:** 10.1016/j.heliyon.2022.e11912

**Published:** 2022-11-29

**Authors:** Belachew Desalegn, Desta Gebeyehu, Bimrew Tamrat

**Affiliations:** a Energy Center, Faculty of Electrical and Computer Engineering, Bahir Dar Institute of Technology, Bahir Dar University, P. O. Box 26, Bahir Dar, Ethiopia; b Department of Physics, College of Natural and Computational Science, Wolaita Sodo University, P. O. Box 138, Wolaita Sodo, Ethiopia; c Department of Mechanical Engineering, Faculty of Engineering, Addis Ababa University, Addis Ababa, Ethiopia

**Keywords:** DFIG WECS, Linear operating behavior, Nonlinear operating behavior, Voltage dip, Indirect field control strategy, PI controller (2DOF)

## Abstract

Owing to different stochastic characteristics of wind energy systems, there would commonly be uncertainties in the processes of wind energy conversion that may ultimately cause to severely degrade the quality of electric power production. These uncertainties include time-varying fluctuations of mechanical & electrical parameters that can be generated during both linear, and nonlinear operating behaviors of doubly fed induction generator-based wind energy conversion system (DFIG WECS). In order to handle a wind power quality problem, the previous studies largely focused on adjustment of mechanical parameters particularly based on blade pitch angle control by proposing different control strategies, and controller models. This work proposes a rarely studied electrical parameter control method that is particularly used to implement the regulation of rotor current components & electromagnetic torque in a DFIG WECS, based on Indirect Field Oriented Control (IFOC) strategy. Accordingly, a novel Proportional Integral controller model that employs a 2-Degree-of-Freedom [PI (2DOF)] is illustrated for an enhanced control of the rotor current components (quadrature & direct currents), electromagnetic torque under a 2MW DFIG WECS, which is operationally assumed to behave both linearly & nonlinearly. Herein, nonlinear operating behavior signifies a voltage dip that was assumed to be resulting when the system's normal (linear) voltage would suddenly drop by 90%. Furthermore, the overall model of the DFIG system was simulated in MATLAB-SIMULINK environment to evaluate the performances of PI controller (2DOF) under the system's stated operating behaviors. Based on the simulation signal statistics, the quadrature current distortion levels & DC mean values were mainly considered as the criteria for evaluating the controller performances. Finally, the proposed PI controller (2DOF) model has been tested to achieve an enhanced power quality in comparison with the traditional PI controller model.

## 1 Introduction

Doubly-fed induction generators (DFIGs) represent the electric power machines that are designed to be fed ac currents into the windings of their stator and rotor correspondently. One of the unique advantages that the DFIGs offer for application in WECS is that their output voltages can be made flexible for power operation by being able to be maintained at constant amplitude and frequency regardless of the quality of wind speed blowing on the WECS rotors [1]. This unique operating feature of DFIGs allow them to be directly linked to the ac power supply by being remaining regularly synchronized according to the power management system. Besides, the better flexibility in the controllability of power factor by maintaining the moderate size of power electrics devices is another special operating characteristics of DFIGs that make them largely preferable for application in WECS [2].

The DFIG WECS is usually modeled with a back-to-back converters, the rotor side converter (RSC) and grid side converter (GSC). A dc link capacitor that serves as energy storage device is placed between these two converters in order to ensure the regulation of voltage ripples in the system. The machine-side converter is used to regulate the electromagnetic torque, the rotor speed, and also the stator power factor at its terminals, whereas the primary function of the grid-side converter is to maintain the operation of dc-link capacitor at constant voltage source [3, 4]. The wound-rotor induction machines are typically implemented in DFIG-based WECSs for ensuring conversion of electromechanical energy [5]. The configuration of DFIG machine is conventionally designed by enabling the connection of the stator terminals to the power grid, and the rotor terminals to a power electronic converter [6].

The operation of a wound-rotor induction machine as a DFIG system can be achieved by considering and implementing the inclusion of a power electronic converter into the rotor circuit. The function of this converter is to direct the power transfer into and out of the windings of rotor so as to support the possibility that the DFIG machine can operate as both motor and generator with two options of rotor operating speeds: sub-synchronous and hyper-synchronous modes. Sub-synchronous operating mode indicates the condition that when the machine runs below synchronous speed, and hyper-synchronous mode of operation is attained under the condition at which the machine tends to accelerate above its synchronous speed [7]. Under both modes of operation, the machine can function as either a generator – where the generated torque is assumed to have negative value, as the machine needs mechanical torque as an input during this operating mode; or a motor – at which the produced torque is conventionally taken to be positive [8].

Numerous studies [9, 10, 11, 12] in recent days have given due attention to the DFIG WECS justifying that its outstanding merits make it to be one of the most desirable generator systems for application in wind energy conversion. It has been in use for many years now in harvesting wind energy, and it has also a significant share of the global commercial market nowadays. Moreover, the leading global cumulative wind electricity installed with DFIG machines has proved that this technology remains of great interest in the wind farm industry though the advanced technologies including the direct drive (gearless) permanent magnet synchronous generator (PMSG) have quite recently come up to gaining an increased consideration by researchers.

More advantages of DFIG-based WECS have generally been detailed in many studies, mainly including: its higher wind energy harvesting capability even under the condition that the wind blows at lower speeds, the flexibility in the independent control of its real power and reactive power – which ensures rapid power response based on the system needs [13], its reduced maintenance costs and extended lifetime due to its minimized mechanical stresses [14], the achievability of power quality improvement with no need to implementing the installation of external reactive compensation devices [15], its power electronic converter (PEC) design is usually rated only for 30% of the power scale – this significantly contributing to the lowering cost of DFIG WECS [16, 17]; and etc. On the other hand, several limitations of DFIG WECS were also widely identified by studies with a special focus on enhancing its power generating reliability [18, 19, 20, 21, 22]. Due to the direct connection of the stator to the grid, the DFIG machine is usually left highly vulnerable to power system disturbances that are mainly resulting from voltage dips, and voltage swells; while voltage dips were proposed to be the most common problem in recent research studies. Since the DFIG system is conventionally based on the partially rated converter, its capability to mitigate rising currents and voltages that are generated in it by stator fault currents as the result of the magnetic coupling between the stator and the rotor was reported to be largely limited.

Meanwhile, many different control strategies along with controller models were proposed by researchers to enhance the power control systems for DFIG WECS by improving its fault ride through (FRT) performances. The improvements involve enhancing the low voltage ride through (LVRT) & high voltage ride through (HVRT) capabilities in order to alleviate serious impacts of voltage dips & voltage swells that could possibly result in power quality disturbances & failures of power systems’ component devices including power electronics. Enhancing FRT performances, by making use of different control strategies & controller models, are ultimately important for the smooth-running of the active power, and reactive power generations by DFIG systems [23, 24, 25]. Hence, FRT performances can be generally improved by implementing a direct or indirect control of active power, and reactive power generations based on various techniques. For instance, a direct field oriented control technique associated with the conventional PI controllers for rotor side and grid side control was independently implemented in [1] with an objective of improving FRT capability, and the desired improvement was reported to be made.

Further, a novel computational intelligence-based control strategy that employs genetic algorithm [26] was proposed to enhance LVRT capability of a grid connected 1.5MW DFIG WECS without a use of any auxiliary hardware, and its effectives was reported to be validated. Additionally, a direct field control technique-based intelligent proportional-integral sliding controller [27] was modeled to improve the stability of power production with 5MW DFIG WECS under several disturbance factors, and the proposed model was demonstrated to outperform the traditional PI controller model. Moreover, indirect field control technique-based comparative study that uses conventional PI, fuzzy and fuzzy-PI controllers was conducted in [8] through control system model simulation of 2MW power rated DFIG WECS by applying MATLAB-SIMULINK software environment; and steady state performance with each controller design was finally analyzed to show the better efficiency fuzzy-PI controller model. The performances of direct, and indirect field oriented control techniques were also comparatively evaluated by some studies. For instance, the Maximum Power Point Tracking (MPPT) strategies based on both direct, and indirect field orientation frames, and by employing conventional PI controllers were comparatively studied with the DFIG WECS by [28, 29]; the results of these studies ultimately indicated that indirect field oriented control technique has generally a better tracking performance. In conclusion, the most recent studies on power systems [30, 31, 32, 33] reiterated that optimal automatic control of electric production is crucial to optimize the overall cost & efficiency of energy extraction including the wind energy development.

### 1.1 Study gap and motivation

The studies summarized under the literature review (Section 1) generally indicate that wind power production can be enhanced by making use of different controller models. As it can be clear from these summaries, the application of different controller models may result in varying scales of achievements of wind power control objectives that include maximizing power production with reducing overall cost of wind energy. Hence, the selection of capable controller models is required to be made in order to successfully achieve these objectives. Indeed, each controller model has its own advantages, and limitations in handling a control subsystem under the varying operating behaviors of a power system, and maintaining the power production over the power system's broad operation range is one of the critical research & technological issues nowadays. A large number of studies including [34, 35, 36] reported on the robustness of traditional PI controller model in capturing wind power that is limited to only over the DFIG WECS's normal (linear) operation range. However, as it has been already mentioned through this section, DFIG WECS would also largely experience nonlinear operating behavior that could be resulting from the voltage variations, namely: voltage dips, and voltage swells. Consequently, this study implements a novel PI controller design model that employs a set-point control optimization in addition to the control gain values for tuning a DFIG WECS's parameters based on the Rotor Side Converter (RSC) control method. Since this advanced controller model operates with two degree of freedom in tuning control loop parameters, it is commonly named as PI controller (2DOF). Unlike the traditional PI controller, a PI controller (2DOF) is characterized by its capability of rapid power disturbance rejection without resulting in significant increase of overshoot in set-point tracking, which makes it an excellent choice for the broader range of application. This controller model is also desirable to alleviate the impact of changes in the reference signal on the control signal. Hence, this novelty of PI controller (2DOF) model can lead to a significant enhancement of DFIG system.

### 1.2 Contribution of the study

One purpose of this study is to comparatively evaluate and quantify the performances of PI controller (2DOF) against that of traditional PI controller by considering both the linear, and nonlinear operating behaviors of DFIG WECS rated with 2 MW based on the implementation of indirect field control technique on RSC. Further purpose is to discuss the results based on the recommended practice & requirements for power harmonic control in the modern electric power systems. The contribution of this work can be interpreted in relation to the research & technological developments that are currently taking place in the field of sustainable energy engineering in general, and wind power systems engineering in particular. As the strategy pursued by this study relies on the machine learning approach of enhancing power generation system, it obviously constitutes one of the most compelling research issues that will be expected to undergo further advances in the future. Hence, as authors, we believe that this work will inspire further studies to be undertaken in the future more than just reporting provisional results.

### 1.3 Paper organization

The next sections of this paper are organized to cover the rest segments of this study. Accordingly, Section 2 presents on the DFIG WECS's mechanical & electrical systems modeling methods, and control techniques based on various aspects. Further, this section demonstrates control loop configurations for the indirect field control technique, and PI controller (2DOF). Section 2 also proposes desirable values for the parameters (gains & set-point weights) of the controller model. The overall model of the proposed system is finally simulated in MATLAB-SIMULINK environment by employing different mechanical and electrical blocks, which is illustrated under Section 3. In addition, based on the simulation, extensive results and discussion that incorporate figures, tables, and analysis for control signals including rotor current components & electromagnetic torque are presented under Section 4. In the end, conclusion of this work is indicated under Section 5 by incorporating future research prospects.

## 2 Methods of power control for DFIG-based wind energy conversion system

**Figure 1:**
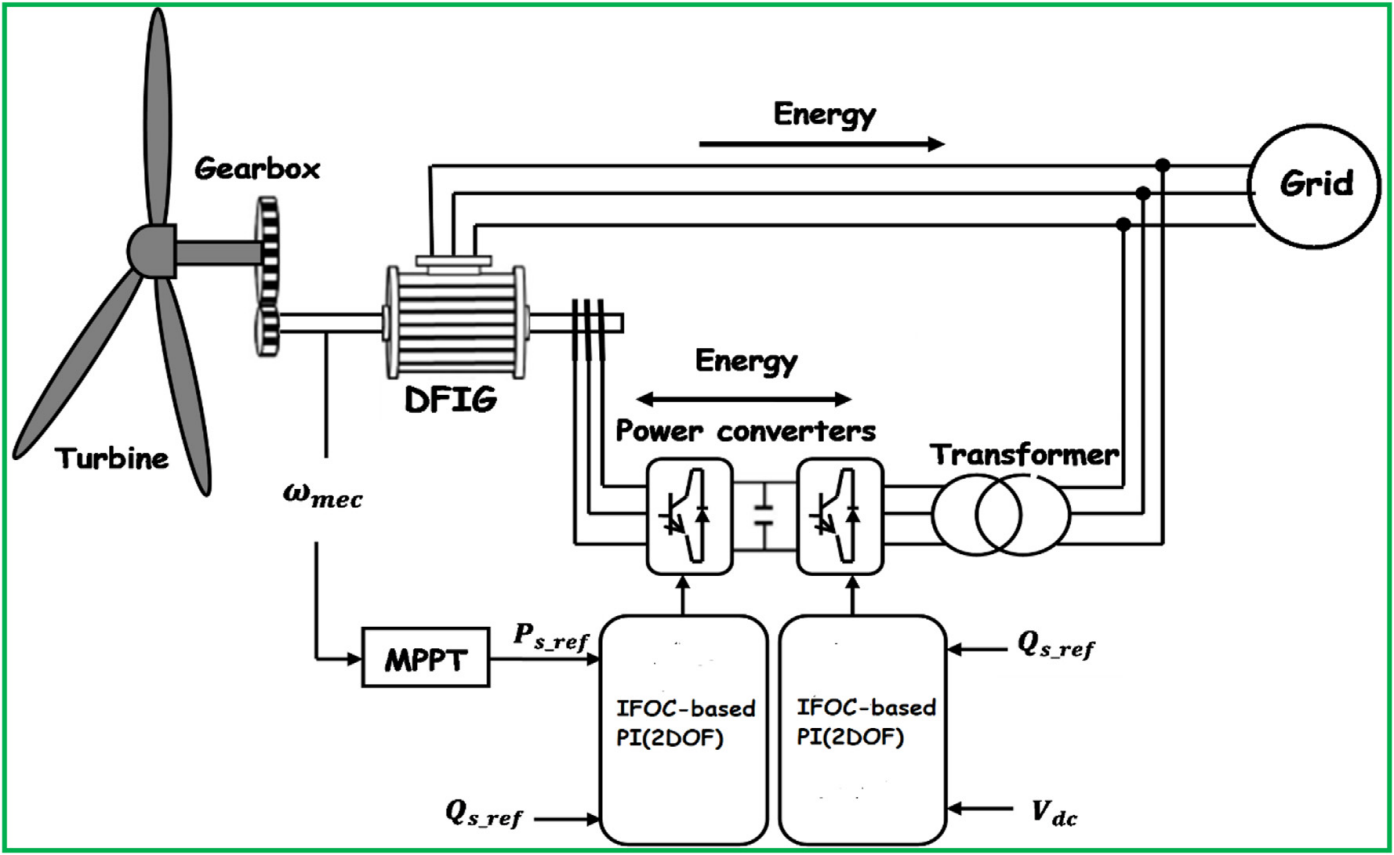
General Configuration of proposed DFIG-based WECS.

A grid connected DFIG WECS that comprises turbine of three blades, a gearbox system, and electric generator, partial power converter system, and step-up transformer is proposed by this study as its overall configuration is shown by Figure 1. The turbine is tied to the DFIG by means of the gearbox to adapt the slow speed of the turbine shaft to the speed of the machine. The generator stator is straightly coupled to grid while the rotor is connected thereto through power converter. Two levels of control system can be distinguished, the rotor side converter (RSC) allows regulating the stator reactive and real power flows. This last one is delivered from the MPPT technique. The grid side converter (GSC) regulates the DC voltage link and the reactive power of the rotor. Only RSC control is considered in this work. In this work, indirect field-oriented control (IFOC) with PI controller (2DOF) is proposed to control the DFIG WECS in variable wind speed mode. More details are covered under subsections (2.1–2.3) to follow.

### 2.1 Control strategy for the mechanical component

**Figure 2:**
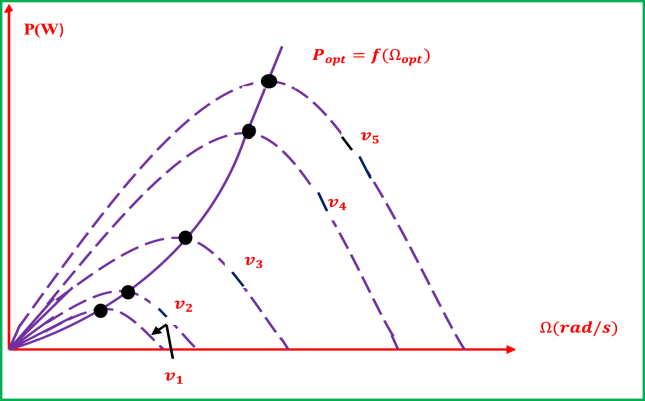
Wind turbine operational characteristics in the plane (power vs. turbine's rotational speed).

A wind turbine's optimal power is characterized to be largely non-linear – demonstrating bell-shaped distribution. The maximum power should be captured with the wind energy conversion system for all wind speed ranges, which would require the optimum rotational speed to be attained. This is illustrated by Figure 2 such that power and rotational speed of the wind turbine follow the wind speed characteristic curves, and each curve correlates with a wind speed $V_{v}\text{.}$ The expected optimal points are resulted from the vertices of these characteristics, and the optimal power curve is mathematically represented by Eq. (1) [37]:

(1) $$P_{Opt}=C_{POpt}\left(\lambda_{Opt}\right)\times \frac{\rho \times \pi \times R^{2}\times V^{3}}{2}$$

A unique command named maximum power point tracking (MPPT) is required for the wind energy conversion system to be able to perfectly track the optimum power curve in order to attain its ideal operation. This strategy can effectively help to maximize the electrical power generation across various wind speed ranges by implementing the control of electromagnetic torque along with adjusting the system's mechanical speed [38].

This strategy can be implemented by employing two methods: one method pursues that the characteristic $C_{P}$ = *f* (*λ*) is not determined, whereas the second method propounds that the characteristic $C_{P}$ = *f* (*λ*) is determined. The second method would help to develop control system with reduced design complexity in enabling to track the ideal power curve such that the wind turbine attains ideal operations. In addition, besides its special advantage in helping to simplify the algorithm of the maximum power point tracking (MPPT), this method can be implemented with application of widely available and cost-effective converters [39].

**Figure 3:**
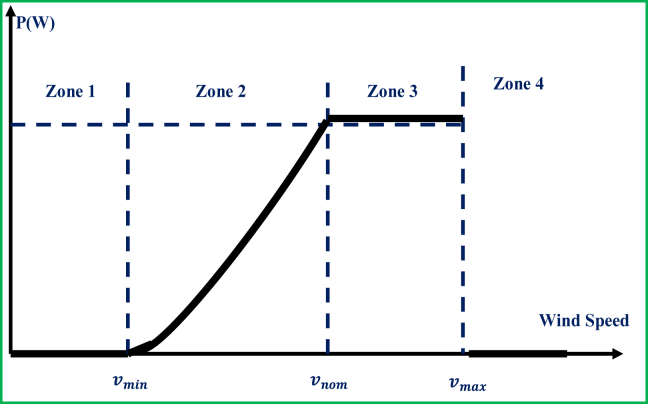
Turbine characteristics based on power vs. wind speed.

The variable-speed wind turbine has four operating phases and these phases can be illustrated according to Figure 3 in addition to the following outlines [40]:

I. The quality of wind speeds is generally too low to be able to sufficiently run a wind turbine for power production.
II. The electromagnetic torque regulation would be implemented, where the wedge angle is fixed, and MPPT principle would be applied in order to ensure the capturing of maximum power for all ranges of wind speeds. Under this phase, a rapid progression associated with the generator power curve is kept.
III. The speed of the generator would be kept constant at its peak value as opposed to a desirable torque. The wind speed increment results in the coefficient $C_{P}$ reduction and the recovered power would slowly get maximized. When the power production is reached its maximum, the coefficient $C_{P}$ would be degraded with the adjustment of the blade (pitch) angles (changing from $\beta_{1}$ to $\beta_{2}$).
IV. Under this phase, the wind speed gets too high ${\;V}_{M}$ and this may cause severe failure to the wind turbine devices that would result in no electricity production. To prevent the damage, an emergency device is used to shut-down the operation of the turbine.

For the extraction of maximum power with the wind turbine, the algorithm that would be acting on the set point variables is required, which would ensure to develop the device with a good efficiency. In the recent literatures, for the maximization of power extraction, two types of control structures were mainly presented:

I. Non-mechanical speed control-based MPPT control.
II. Mechanical speed control-based MPPT control.

In this work, the MPPT control that is based on non-mechanical speed control is proposed, because achieving an accurate measurement of wind speed would be difficult due to the facts that are outlined as follows [41]:

⁃ The anemometer would be mounted at the space behind the wind turbine's rotor, and this would lead to an inaccurate wind speed measurement.
⁃ A considerable difference in wind speed results based on the height at which the anemometer is mounted due to the reason that the diameter of the surface swept by the blades is extensive, which is typically 70 m for a wind turbine of 1.5 m. Hence, the implementation of a single anemometer would lead to the usage of only one local estimation of the wind speed, which is obviously not sufficiently representing the wind speed average value appearing across the entire blades.

**Figure 4:**
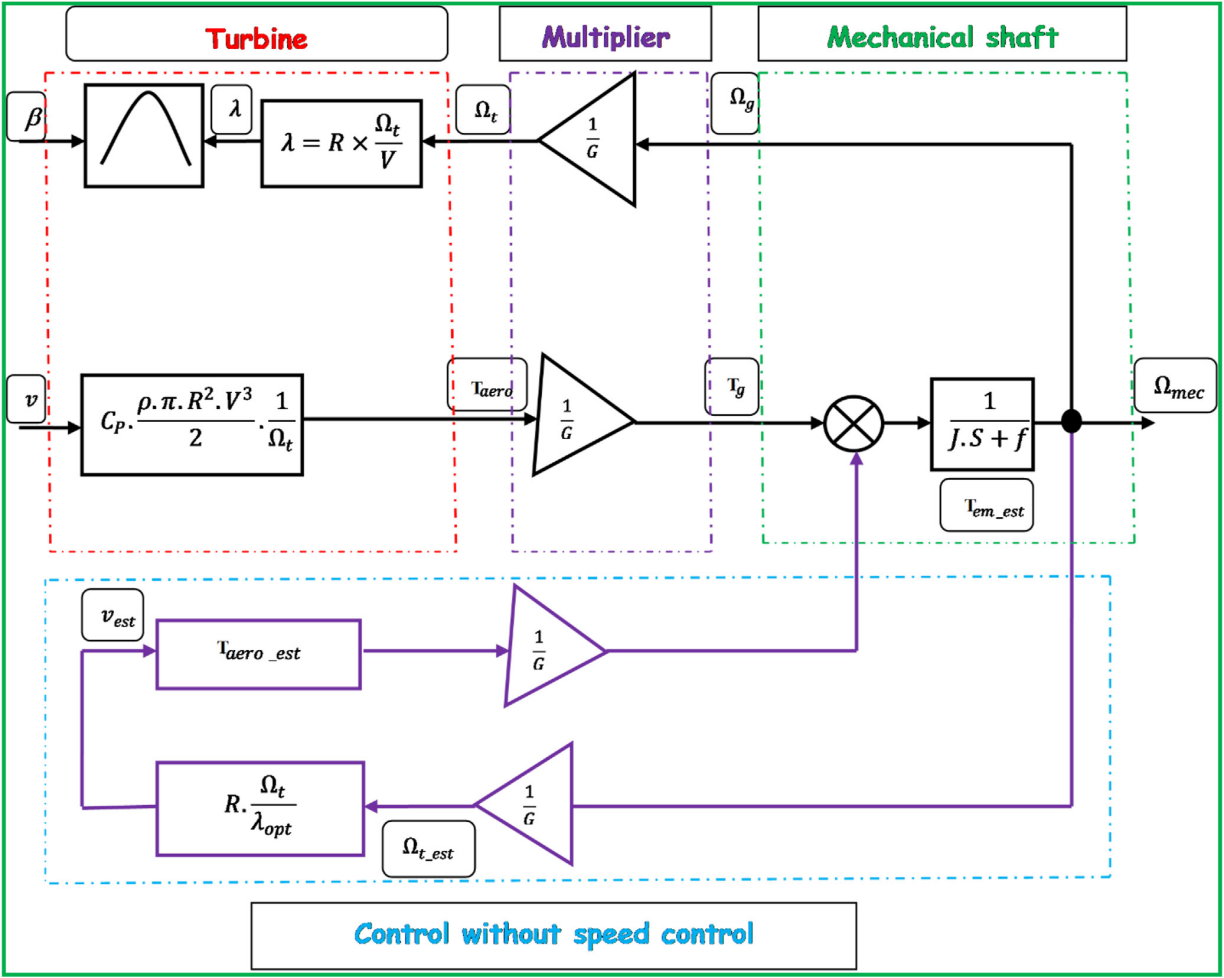
Block diagram of non-speed control-based maximum power point tracking (MPPT) strategy.

In general, an inaccurate estimation of the wind speed would inevitably result in a degradation of power that could be harnessed according to the technique of wind energy extraction presented above. Quite recently, an increasing number of wind turbines are controlled without the implementation of mechanical speed control, and the wind speed is considered to be varying very steadily according to this control structure (its block diagram is illustrated by Figure 4). In addition, Eq. (2) represents the strategy of non-mechanical speed control-based MPPT control, which is written as:

(2) $$J\frac{d\Omega_{mec}}{dt}=T_{g}-T_{em}-f\times \Omega_{mec}=0$$

With the omission of the mechanical torque $C_{mec}$ and the effect of the couple of viscous friction $f\times \Omega_{mec}$, Eq. (2) can be simplified as follows [Eq. (3)]:

(3) $$T_{em}=T_{g}$$

The reference electromagnetic torque can be determined based on an estimation just as defined by the following mathematical expressions [Eqs. (4), (5), and (6)]:

(4) $$T_{emref}=\frac{T_{aeroref}}{G}$$

Moreover, aerodynamic torque $C_{aero}$, turbine angular speed $\Omega_{t}$ and estimated wind speed $V_{est}$ can be quantified according to the following equation:

(5) $$\left\{ \begin{array}{l} T_{aeroref}=C_{P}\left(\lambda ,\beta \right)\frac{\rho \times \pi \times R^{2}\times V^{3}}{2\times \Omega_{t}}\\ \Omega_{t}=\frac{\Omega_{mec}}{G}\\ V_{est}=R\frac{\Omega_{test}}{2\times \lambda_{test}} \end{array}\right.$$

With the series of substitutions and rearrangements, the reference electromagnetic torque $C_{emref\;}$ can be redefined by Eq. (6):

(6) $$T_{emref}=\frac{C_{P}\left(\lambda ,\beta \right)}{\lambda_{opt}^{3}}\times \frac{\rho \times \pi \times R^{3}}{2}\times \frac{\Omega_{mec}^{3}}{G^{3}}$$

And more alternatively, the aerodynamic torque reference can be expressed in terms of its coefficient $C_{t}$ based on Eq. (7) [42], as follows:

(7) $$T_{aeroref}=\frac{1}{2}\rho \pi R^{3}V^{2}C_{t},where\;C_{t}=\frac{C_{Popt}}{\lambda_{opt}}$$

Hence, the mathematical expression for electromagnetic torque reference can be remodified by applying Eq. (4) and Eq. (7) to take the following form [Eq. (8)]:

(8) $$T_{emref}=\frac{\rho \pi R^{3}V^{2}C_{Popt}}{2\lambda_{opt}G}$$

### 2.2 Control strategy for the electrical machine

**Figure 5:**
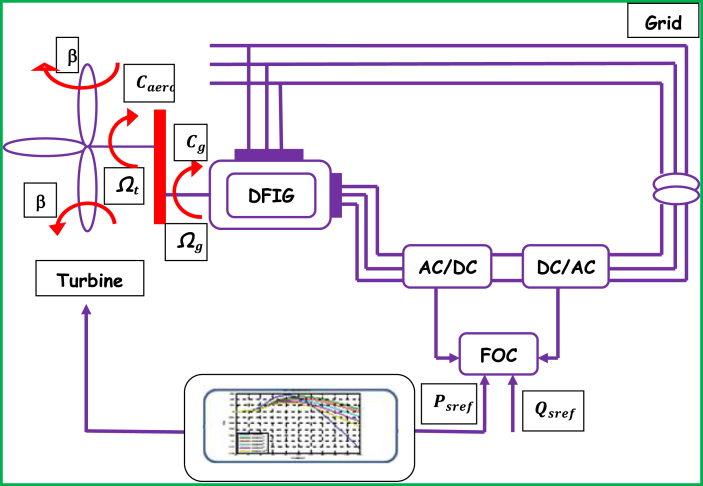
Universal configuration of DFIG-based wind energy conversion system (WECS).

This study proposes the wind energy conversion system that is based on the illustration by Figure 5. Two main components of this system are considered to be modeled separately as follows: the stator of doubly fed induction generator (DFIG) is connected to the grid in a direct configuration, whereas its rotor is connected to the grid through power converters in back-to-back (BTB) configuration. These converters have the characteristics to operate as both rectifier and inverter based on the direction of the energy transport.

In order to implement the power control strategies with the objective of maintaining the stability and quality of electricity production by regulating the power transfer between the generator and the grid, the configuration of the electrical machine demonstrated by Figure 5 is retained in this study. The reason that the power converters are not required to be used for transferring of the stator power is that the stator magnitudes have frequency which exactly equals that of the grid. In contrast, the rotor and the grid operate at magnitudes that alternate with different frequencies. Hence, the rotor power largely relies on the speed of the rotor and indeed on the wind speed, and this necessitates a voltage converter application for supporting the rotor power so that it would get to alternate at the same frequency with that of the grid. The DFIG-based wind energy conversion system can be designed as constant or variable-speed applications, and hypo-synchronous or hyper-synchronous operation modes under the context of electricity generation from wind energy. The rotor side power converter enhances the regulation of active and reactive power production, whereas the power converter at grid side reinforces the control of the DC bus voltage and the grid-power factor. The application of DFIG technology in wind farms can exhibit an outperforming capability since it presents a unique compromise between its range of speed variation and the dimensioning of the power converters against its nominal power.

**Figure 6:**
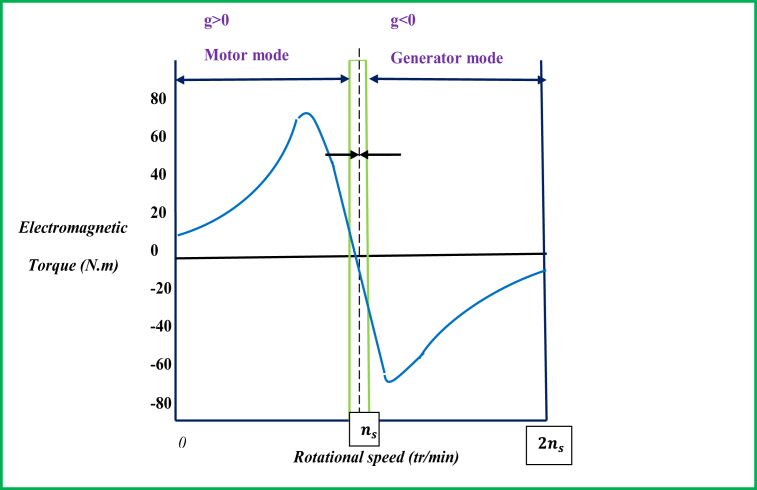
Torque/speed operating condition of DFIG machine.

Unlike other electrical machines, the DFIG machine is reversible and designed for both generator and motor operation modes. For specific discussion here, the mechanical torque/speed characteristic-based mode of operation is shown by Figure 6 for the induction machine. As it can be observed from this figure, the induction machine exhibits motor operation mode when *g >* 0 and generator's mode of operation in the reverse direction. In the context of DFIM's operation, the stator is inverted by a first balanced 3-phase voltage source with frequency *f,* the rotor is tied to a second alternating frequency source $f_{r}\text{.}$ The currents that are flowing in the stator windings induce a stator field rotating at the speed $\Omega_{s}=\frac{\Omega_{s}}{P}\text{,}$ and likewise, the rotor currents create a rotating rotor field at a speed $\Omega_{r}=\frac{\Omega_{r}}{P}$. The machine keeps hypo-synchronous mode of operation when the rotor and the stator fields alternate in the same direction, which causes the slip g to have a positive value and the rotor field to rotate with slower speed compared to the stator field ($\Omega <\Omega_{s})$. On the other hand, the machine follows hyper-synchronous-based operation mode under the condition at which the field produced by the rotor windings vibrates in the direction opposing the vibration of the stator field, where the slip g turned into negative value the rotor undergoes faster vibration than the vibrating field generated by stator windings ($\Omega >\Omega_{s})$.

The convention that sounds as: ‘the slip's positive sign signifies a motor operation mode of the machine, whereas a negative sign corresponds to a generator operation mode’ is applicable for the conventional induction machine, but it poses confusion to comprehend the operating principle of the DFIM, where the slip's sign indicates hypo or hyper-synchronous operation and not actually reflecting the machine's operating modes, motor or generator. Yet, to ensure the machine to operate in hyper-synchronous or in hypo-synchronous, and both in modes of motor and generator, the control of the amplitude and phase of the rotor voltages has to be implemented. This facilitates the control of the magnetic field at the space inside the machine.

The scale of wind energy extraction would also profoundly depends on the control strategies that could be implemented with different orientation frames in addition to the type of WT generator. A number of control designs have been recently introduced by researchers for studying the characteristics of DFIG-based WECS during normal and faulty grid (AC) conditions. The most prominent technique relies on the field oriented control (FOC) strategy and it is implemented for independently controlling active and reactive power drawn from the supply.

Stationary and synchronous frames-referenced control schemes are developed in this study. The reference frame models for DFIM have been also developed. Algorithms are written for these methodologies in relating firing sequence (for firing back-to-back converters) to DFIG output parameters including active and reactive power via FOC strategy. Moreover, the control of DFIG is implemented by employing FOC in order to ensure the decoupling of the rotor currents into active power/torque and reactive power/flux components and separately adjust them in a reference frame oriented to either stator's flux or voltage. The rotor currents are eventually regulated by utilizing the current controllers.

As it has been already indicated, this study is based on the modeling and control of the WECS that use the DFIG technology with regulation of active and reactive power by utilizing the MPPT strategy. The output power fluctuations would result in the generation of thermal cycling, and this can severely affect the power electronics converter (PEC) with the effect of failures that are associated to semiconductor device and bond wire connection. In addition, the voltage fluctuations that could be induced with the reducing output power, and the harmonic currents that may be generated with increasing variations in output currents can also affect the PEC's operation with the effect of failures that are particularly associated to the DC-link capacitor. Here, the maximum power extraction with DFIG can be achieved by the implementation of active and reactive power control, which indeed help to ensure enhanced power quality and protected PEC. Besides, the possible and instant occurrences of fall in grid voltage can result in the limitless rising of currents in the rotor windings, and this can threaten the generator's safety on the other hand. Hence, regulation of rotor currents is aimed at keeping the generator's protection in addition to help maximizing power production.

The principle of control by FOC consists in orienting field along one of the axes so as to make the characteristics of the induction machine identical to that of the machine which is DC separately excited.

**Figure 7:**
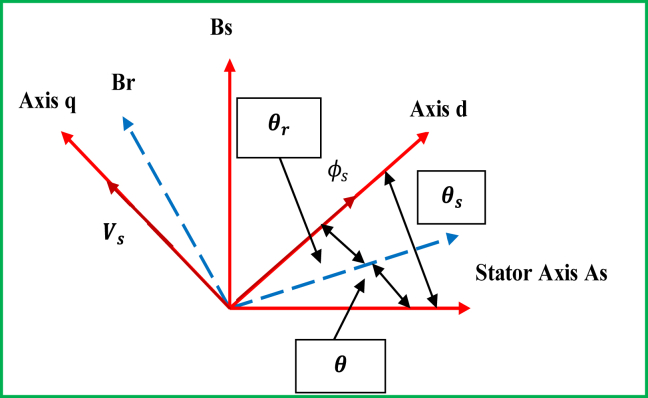
The orientation of stator field across the d-axis.

The stator filed has been considered to be oriented along the axis ‘d’ as shown in Figure 7. On the basis of this orientation, the stator fluxes are defined according to Eq. (9) [43], as follows:

(9) $$\left\{ \begin{array}{l} \Phi_{sd}=\Phi_{s}\\ \Phi_{sq}=0 \end{array}\right.$$

The voltage at the terminals of a phase ‘i’ of the stator (based on the application of Park transformation to DFIG) can be expressed as follows [Eq. (10)]:

(10) $$v_{si}=R_{s}\times I_{si}+\frac{d\Phi_{si}}{dt}$$

With :i=1,2,3.

The resistance of the stator winding ${{\prime R}_{s}}^{\prime }$ can be assumed to be negligible in the cases of the medium and high power machines that are generally employed in the wind power generation, Eq. (10) can be simplified as [Eq. (11)]:

(11) $$v_{si}=\frac{d\Phi_{si}}{dt}$$

By supposing that the electrical grid voltages are stable, and the stator flux also gets fixed; the stator voltages can be given as follows [Eq. (12)]:

(12) $$\left\{ \begin{array}{l} V_{sd}=0\\ V_{sq}=V_{s}=\omega_{s}\times \Phi_{s} \end{array}\right.$$

Similarly, the stator flux has been chosen to be oriented across the axis ‘d’ in defining the stator (direct and quadrature) currents according to Eq. (13):

(13) $$\left\{ \begin{array}{l} I_{sd}=\frac{1}{L_{s}}\times \left(\Phi_{s}-M\times I_{rd}\right)\\ I_{sq}=-\frac{M}{L_{s}}\times I_{rq} \end{array}\right.$$

On the other hand, the relationship between the electromagnetic torque and rotor (quadrature) current can be demonstrated in terms of the mathematical expression for the torque [Eq. (14)]:

(14) $$T_{em}=p\times I_{sq}\times \Phi_{s}=-p\times \frac{M}{L_{s}}\times \Phi_{s}\times I_{rq}$$

The relationship between stator's powers (active and reactive) and rotor currents can also be established by utilizing the expressions for $I_{sd}$ and $I_{sq}$ in Eq. (13), and recognizing that $v_{sd}=0\text{.}$ Hence, the expression for the active power ($P_{s})$ and reactive power $\left(Q_{s}\right)$ can be put as [Eq. (15)]:

(15) $$\left\{ \begin{array}{l} P_{s}=V_{sq}\times I_{sq}=-V_{s}\times \frac{M}{L_{s}}\times I_{rq}=-\omega_{s}\times \Phi_{s}\times \frac{M}{L_{s}}\times I_{rq}=\frac{\omega_{s}}{p}T_{em}\\ Q_{s}=V_{s}\times I_{sd}=\frac{V_{s}^{2}}{\omega_{s}\times L_{s}}-V_{s}\times \frac{M}{L_{s}}\times I_{rd} \end{array}\right.$$

With $\frac{\omega_{s}}{p}=\omega_{r}\text{,}$ the most simplified expression for the stator power can be given as [Eq. (16)]:

(16) $$P_{s}=\omega_{r}T_{em}$$

To determine the relationship between rotor voltages (direct and quadrature) and rotor currents (direct and quadrature), the rotor fluxes (${Փ}_{rd}$ and ${Փ}_{rq})$ need to be first defined by employing the expressions for $I_{sd}$ and $I_{sq}$ in Eq. (13) again. According, the fluxes take the following forms [Eq. (17)]:

(17) $$\left\{ \begin{array}{l} \Phi_{rd}=\left(L_{r}-\frac{M^{2}}{L_{s}}\right)\times I_{rd}+\frac{M\times v_{s}}{L_{s}\times \omega_{s}}\\ \Phi_{rq}=\left(L_{r}-\frac{M^{2}}{L_{s}}\right)\times I_{rq} \end{array}\right.$$

Now, by applying the expressions for rotor fluxes $({Փ}_{rd}$ and ${Փ}_{rq})$ that are represented by Eq. (17), the rotor voltages in terms of these fluxes, and hence, the dependences of the rotor voltages $(V_{rd}$ and $V_{rq})$ on the rotor currents $(I_{rd}$ and $I_{rq})$ are indicated by the following mathematical expressions for $V_{rd}$ and $V_{rq}$ [Eq. (18)]:

(18) $$\left\{ \begin{array}{l} V_{rd}=\left[R_{r}+S\left(L_{r}-\frac{M^{2}}{L_{s}}\right)\right]I_{rd}-\omega_{s}\times g\left(L_{r}-\frac{M^{2}}{L_{s}}\right)I_{rq}\\ V_{rq}=\left[R_{r}+S\left(L_{r}-\frac{M^{2}}{L_{s}}\right)\right]I_{rq}-\omega_{s}\times g\left(L_{r}-\frac{M^{2}}{L_{s}}\right)I_{rd}+\frac{g\times M\times V_{s}}{L_{s}} \end{array}\right.$$

**Figure 8:**
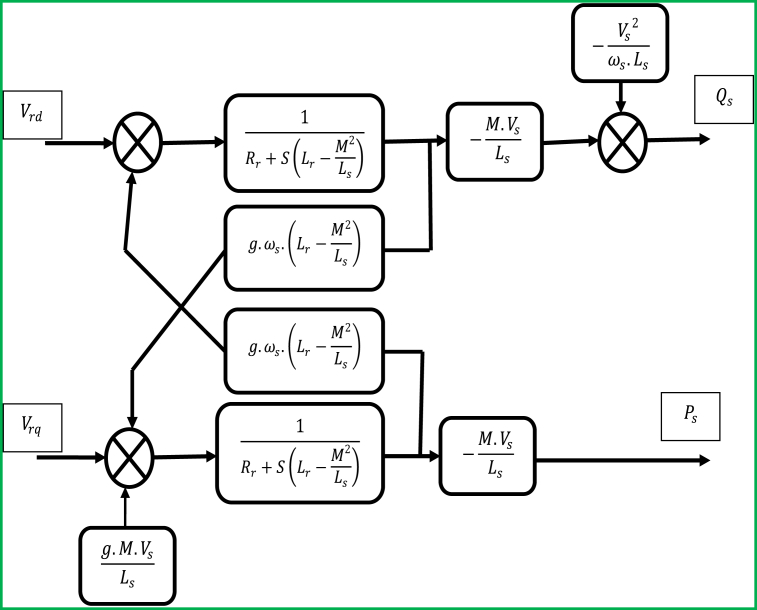
Simplified control model of the DFIG WECS based on Eq. (15) and Eq. (18).

From the above representation (Figure 8), where g is the slip of the induction machine (DFIG), and $\omega_{s}$ is the stator angular speed; $g.\omega_{s}$ is mathematically defined to represent the rotor angular speed $\left(\omega_{r}\right)$ – hence, $\omega_{r}=g.\omega_{s}$. The mathematical expressions for $V_{rd}$ and $V_{rq}$ [Eq. (18)] facilitate the development of a block diagram (Figure 8) for the electrical system that can be regulated by using various controllers. Again, based on this block diagram (Figure 8), the following control premises are set to be considered in developing the control system model:

⁃ The first order transfer functions are used to develop linkage between the rotor voltages $(V_{rd}$ and $V_{rq})$ and the stator powers $(P_{s}$ and ${\;Q}_{s})$. This allows configuring a field (vector) control with the inferences of the couplings, and the independent control can be implemented on each control axis with a controller.
⁃ For a given controller, the reference values are active power and reactive power for the ‘q’ rotor axis and the ‘d’ rotor axis respectively.
⁃ The reference for the reactive power is set to zero, and this ensures maintaining a unit valued power factor on the stator side so that the quality of the power fed to the grid is ultimately optimized. Besides, the active power reference should allow maintaining the optimal value of the wind power factor.

### 2.3 Proposed control configuration for the system development

**Figure 9:**
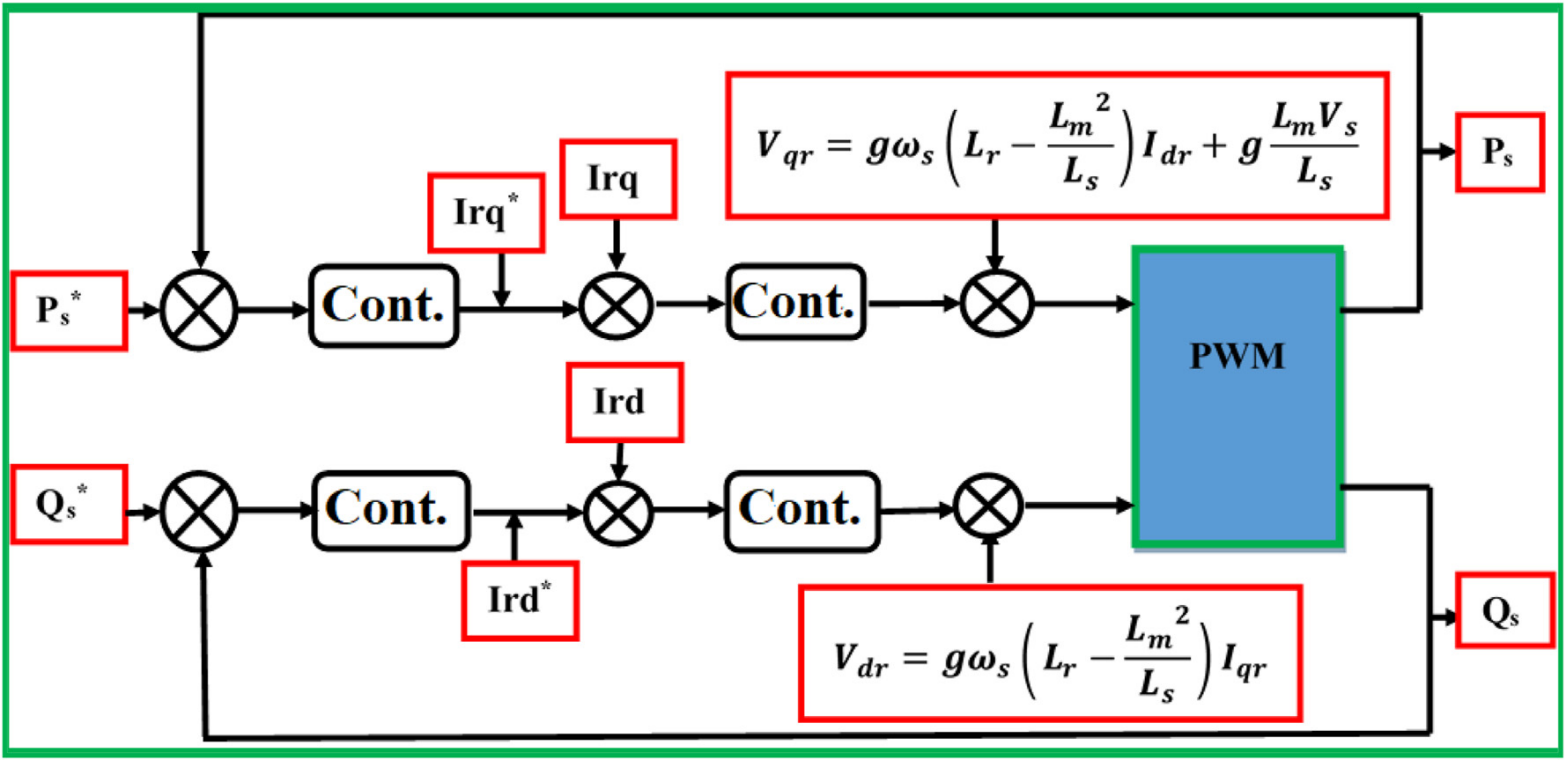
Proposed control structure based on IFOC strategy.

**Figure 10:**
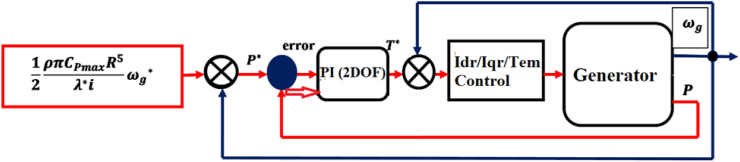
Power control structure for PI controller (2DOF).

On the basis of indirect field oriented control-based maximum electrical power point tracking (IFOC-MEPPT) strategy, the proposed control set up is configured according to a block diagram that is indicated by Figure 9. The 2 level pulse width modulation (2L PWM)-based power control technique that involves the applications of two controller (Cont.) models was employed to implement IFOC-MEPPT strategy in order to regulate the rotor current components, and ultimately control electromagnetic torque of a 2 MW of electricity producing DFIG-based WECS. Eventually, the qualities of stator active ($P_{s}$) & reactive $\left(Q_{s}\right)$ power productions were evaluated based on estimations of total harmonic distortion factors of the rotor quadrature current $\left(I_{qr}\right)$’s signal statistics, and by interpreting the rotor DC current $\left(I_{dr}\right)$ offset values under both linear & nonlinear voltage operating behaviors of the DFIG system. In this study, the voltage values for the DFIG system's linear & nonlinear operating behaviors are specified in Table 1, where the linear voltage value was adopted to be 690 V; and the nonlinear value was assumed to be 69V based on the fact that the system's normal operating voltage may drop by 10%–90% under the power system disturbances. For an ultimate effectiveness of this study, a modified Proportional Integral (PI) controller model that uses a two-degree-of-freedom (2DOF) tuning method is proposed to regulate the rotor current components ($I_{dr}$ and $I_{qr}$), and electromagnetic torque under the aforementioned operating behaviors of the DFIG system. The power control loop structure for this enhanced PI controller model is illustrated by a block diagram of Figure 10. Unlike the conventional PI controller model, the reference & measured control variables in the PI controller (2DOF) model are handled through two input ports to ensure the achievement of enhanced control objectives.

**Table 1:** Proposed control gain & set-point values for PI (2DOF) based on model-based tuning method.

Operating behaviors	Voltages (V)	$K_{Pidr}$	$K_{Iidr}$	$K_{Piqr}$	$K_{Iiqr}$	$K_{PTem}$	$K_{ITem}$	$b_{idr}$	$b_{iqr}$	$b_{Tem}$
Linear	690	0.5771	491.5995	0.5771	491.5995	5080	203200	0	1	0
Nonlinear	69	0.5771	491.5995	0.5771	491.5995	5080	203200	0	0.9	0

Furthermore, the control optimization principle of the proposed PI controller (2DOF) for the rotor current components ($I_{dr}$ and $I_{qr}$), and electromagnetic torque is given according an expression of Eq. (19); such that: $K_{P}$ is a proportional gain, $K_{I}$ is an integral gain, b is a control set-point weight, r−y represents the difference between the reference & measured control parameters, $T_{s}$ is integrator time, and z is discrete time interval. This principle serves to develop a power control system that can ensure to maintain the quality of electric power production under a broader range of the DFIG system's voltage operating behaviors as opposed to the principle of traditional PI controller model, which does not employ an option of control set-point optimization.

(19) $$U_{PI\left(2DOF\right)}=K_{P}\left(b.r-y\right)+K_{I}.T_{s}\frac{1}{z-1}\left(r-y\right)$$

Accordingly, the main purpose of this work is to evaluate the performances of the PI controller (2DOF) in the application of both linear, and nonlinear power production regulation based on a 2 MW power rated-DFIG WECS. The specifications of this system along with an estimated wind speed of 10 m/s, optimum power coefficient of 0.44, aerodynamic torque coefficient of 0.061, optimum tip speed ratio of 7.2, etc. were employed to simulate the overall system model. As a component of the overall DFIG system simulation, the design for PI controller (2DOF) was modelled by applying a built-in PID controller block in MATLAB-SIMULINK environment by implementing the optimum control gains & set-point values that are presented in Table 1. For the rotor current components, the PI controller gains, i.e. $K_{P}$ and $K_{I}$ were tuned until the optimum control values are attained. The optimum control value of $K_{P}$ gain was found to be the same for both current components, direct $\left(I_{dr}\right)$ and quadrature $\left(I_{qr}\right)$ currents, which is: ${\;K}_{P}=0.5771$; and the integral control gain value was also weighting equal for these currents, with: $K_{I}=491.5995$. Besides the regulation of rotor current components, the PI controller (2DOF) model for regulating electromagnetic torque $\left(T_{em}\right)$ was developed to operate as an indirect speed controller, such that: $K_{P}=5080$, $K_{I}$ =203200 were chosen as the controller's optimum gain values.

In addition, to achieve the overall robust control objective for the proposed DFIG system, the PI controller (2DOF) model's optimum control set-point weight (b) values were also proposed to accompany the $K_{P}$, and $K_{I}$ values for the rotor current components ($I_{dr}$ and $I_{qr}$), and electromagnetic torque $\left(T_{em}\right)$. The optimum control set-point weights are generally obtained between & including 0, and 1. Accordingly, the linear optimum control set-point weights were obtained at values of 0, 1, and 0 for rotor direct current $\left(I_{dr}\right)$ controller, quadrature current $\left(I_{qr}\right)$ controller, and electromagnetic torque $\left(T_{em}\right)$ controller respectively. Similarly, the nonlinear optimum control set-point weight values were obtained to be 0, 0.9, and 0.

In the end, the performances of the PI controller (2DOF) were evaluated against that of the traditional PI controller by conducting analyses and discussions particularly based on the quadrature current ($I_{qr}$) harmonic distortion estimation, the DC ($I_{dr}$) reference tracking characteristics, and the scales of produced electric power in association with the generated simulation signal statistics.

## 3 Simulation of control model for a 2 MW power rated DFIG WECS

**Figure 11:**
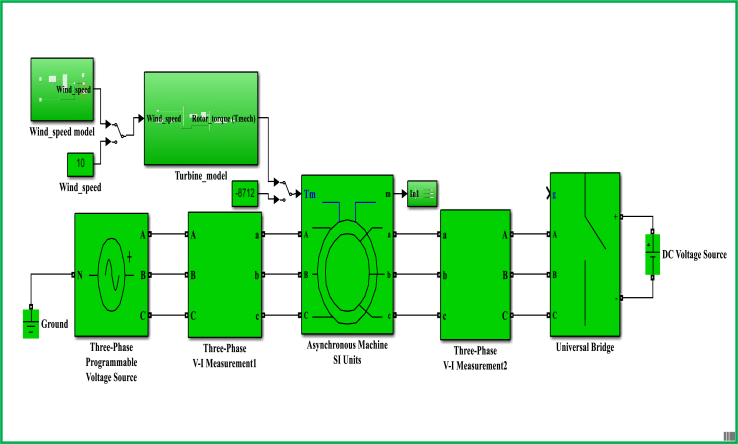
Electrical system design model simulation.

**Figure 12:**
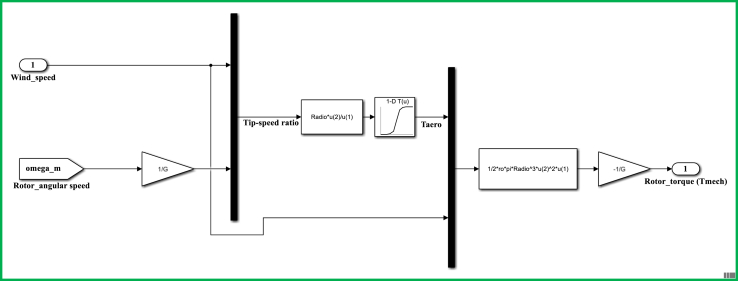
Aerodynamic model simulation.

**Figure 13:**
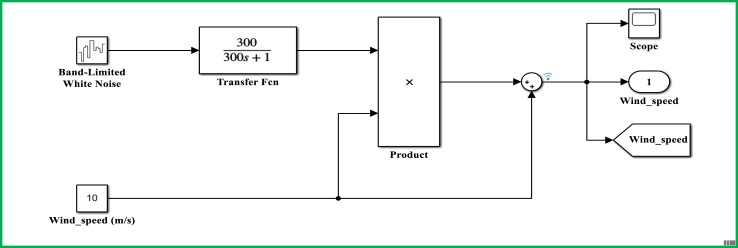
Wind speed model simulation.

**Figure 14:**
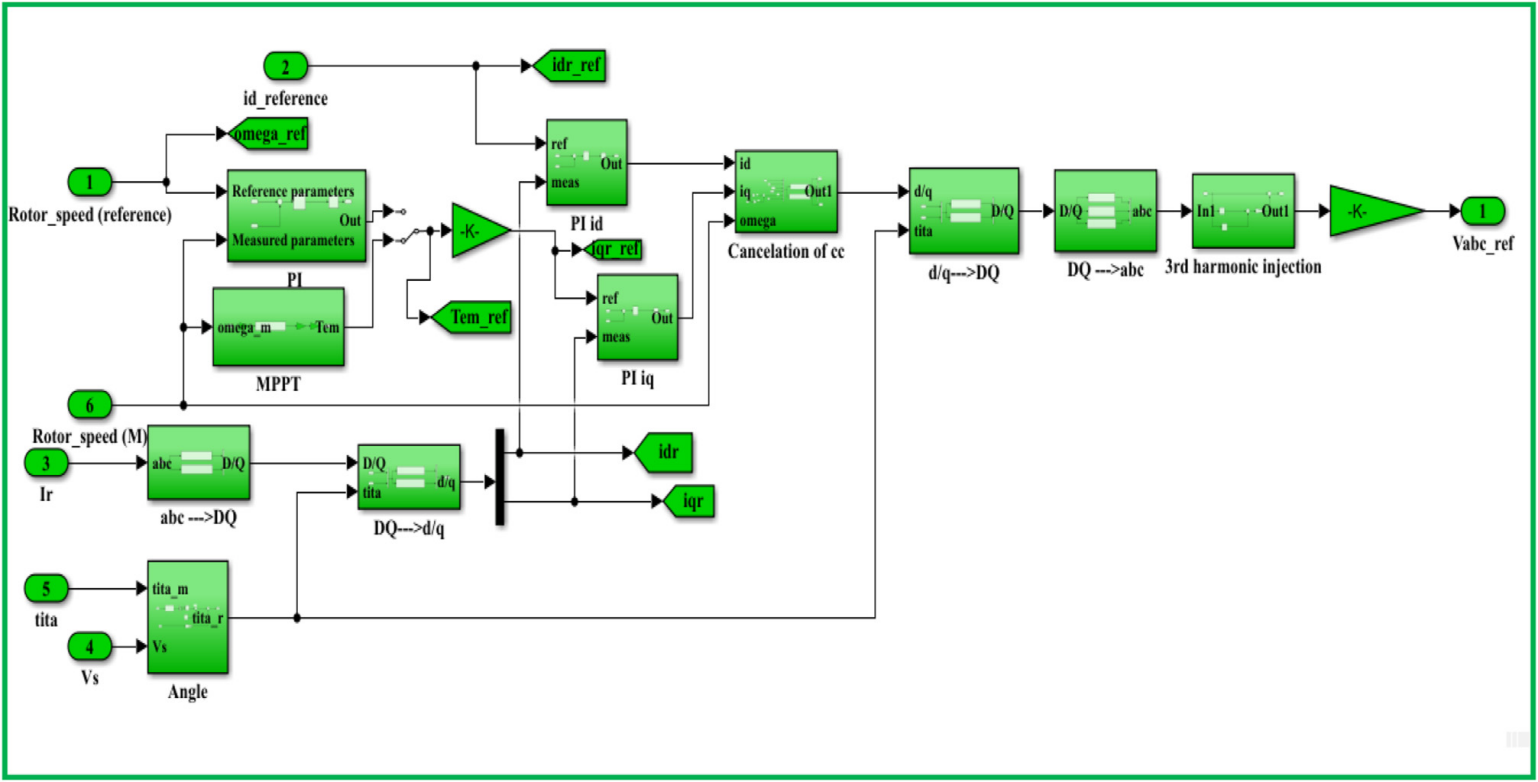
Control system design model simulation.

**Figure 15:**
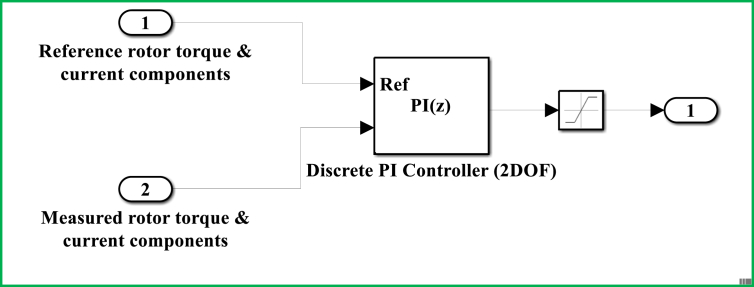
Simulated model for PI controller (2DOF).

The control model for a DFIG WECS of 2 MW rated-power is simulated in the MATLAB-SIMULINK software interface based on the wind speed of 10 m/s, and by making use of different built-in blocks along with the consideration of the system's manufacturer specifications that are presented under Appendix, in Table 5. This control model consists of different subsystems including electrical system design (Figure 11), aerodynamic system simulation (Figure 12), the wind speed model simulation (Figure 13), control system design (Figure 14), and PI controller (2DOF) design (Figure 15). The electrical design model is mainly built by using three-phase programmable voltage source, three-phase V–I measurement, asynchronous machine, and DC voltage source-based universal bridge as SIMULINK blocks. This subsystem is demonstrated in alignment with wind speed model, turbine/aerodynamic model, and mechanical model of the proposed DFIG WECS.

**Figure 16:**
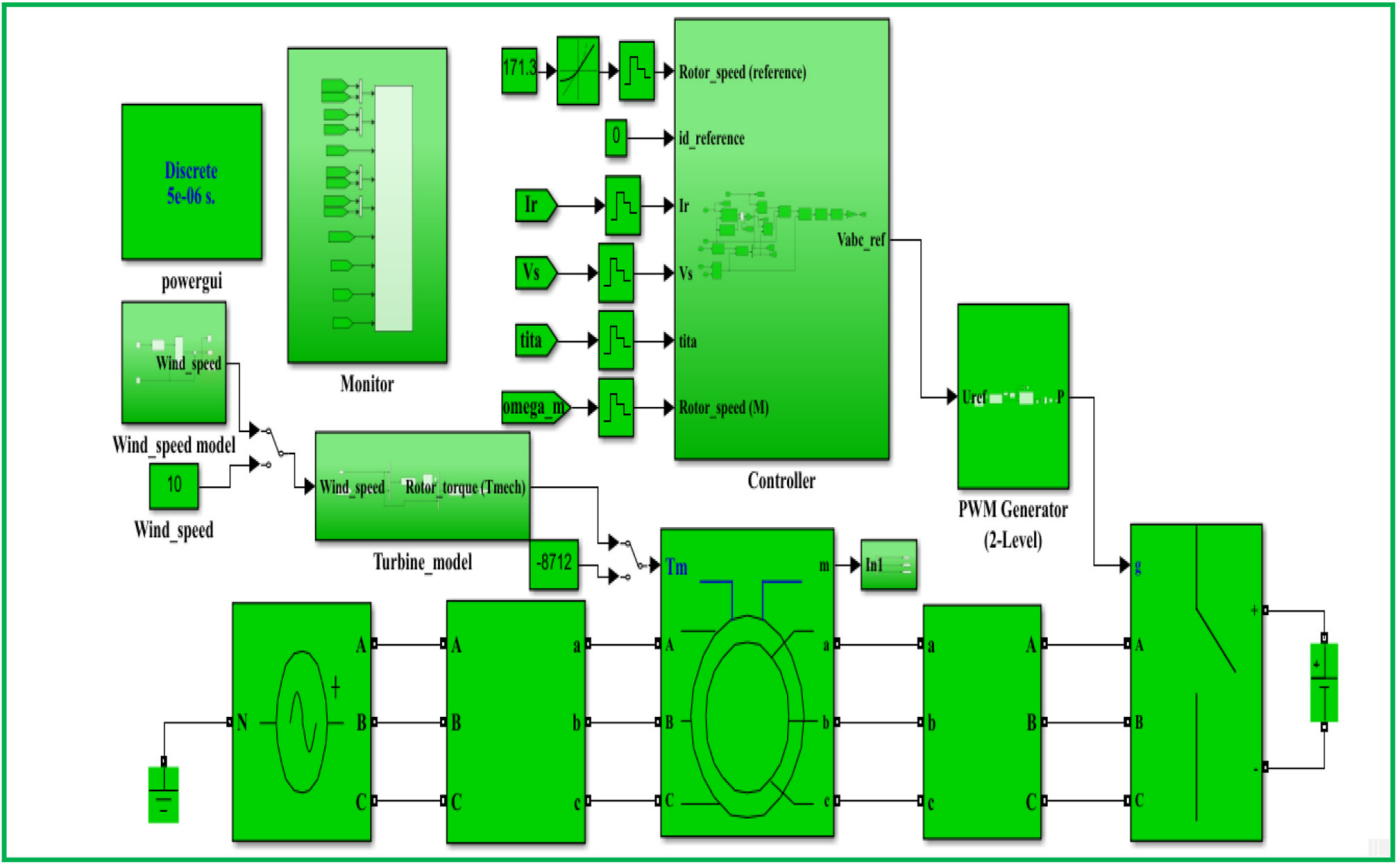
System's overall design model simulation.

The control subsystem model incorporates the PI control design models for the regulations of rotor currents, and electromagnetic torque. It also includes MPPT model, and designed to ensure the transformations of control parameters (including rotor speed, electromagnetic torque, and rotor current components) in accordance with IFOC strategy. The overall system design simulation incorporating electrical system model, control system model, mechanical model, aerodynamic model, wind speed model, PWM generator (2 level), powergui, and control parameters is shown as Figure 16. The function of PWM block is to regulate the amplitude of signals of control parameters (primarily include rotor currents) in order to ensure the protection of system's components particularly generator. Powergui block is employed for the purpose of discretizing the electrical system for running simulation at determined time steps, and in this work, discrete phasor solution is chosen.

## 4 Results and discussion

The purpose of the simulation was to analyze the performances of PI controller (2DOF) under both normal and faulty operating conditions of the DFIG machine. The normal (linear) operating condition is said to be attained when the DFIG system operates within the reasonable control of the specification parameters or when it steadily produces electric power within the specified values of its parameters. On the other hand, faulty (nonlinear) operating condition may occur under the sudden variations of the specified parameters of the machine. Under this condition, the analysis is conducted by assuming that the stator voltage would tend to suddenly fall outside its specified value, which was rated by the machine's manufacturer. Here, the assumption was based on the literatures that the most critical impediment to power quality and stability in the recent days stems from the sudden fluctuations of the grid voltage, which commonly understood as voltage dip – sudden and sharp fall of voltage specification.

**Figure 17:**
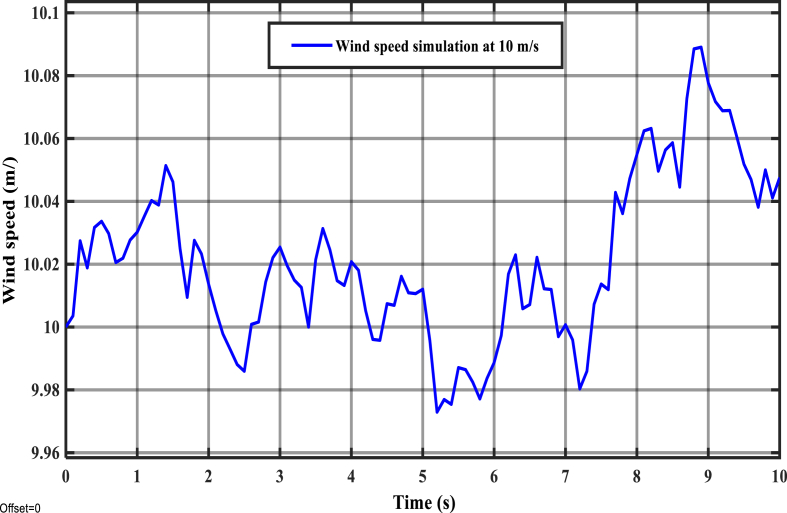
Wind speed simulation result at 10 m/s.

The aforementioned voltage characteristics could particularly cause the system's rotor currents, and electromagnetic torque to be exponentially rising; and this may in turn potentially impact the safety of the system's component devices, and power generation reliability. In principle, WECSs are supposed to operate under faulty conditions as well by maintaining electricity production. Further, the reliability of electric power production, and the protection of electrical component devices should be uncompromisingly ensured by implementing control of the machine's parameters. In this work, the impacts of the rotor currents on the power quality under both normal and faulty operating conditions of the 2 MW DFIG WECS are comparatively studied by employing IFOC-based PI controller (2DOF). Wind speed was randomly simulated at 10 m/s as indicated by Figure 17, and it was used to mathematically compute reference electromagnetic torque ($T_{em\_ref})$.

**Figure 18:**
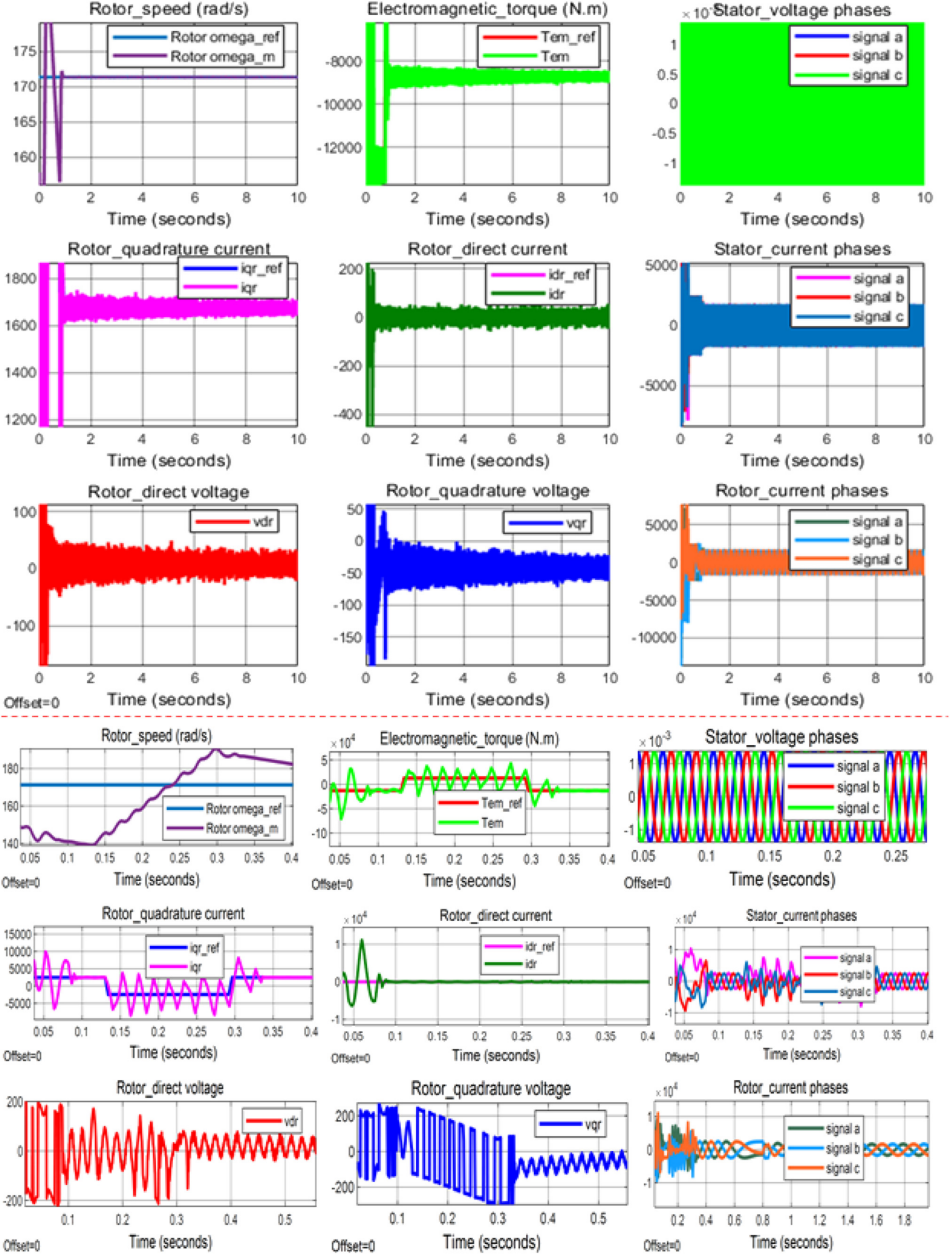
PI (2DOF) performance under system's linear operating condition.

According to the principle of rotor side control (RSC) method, the reference value of direct rotor current (idr_ref) should always be tuned to zero to maintain the indirect regulation of the stator reactive power so that the maximum transfer of power to the electrical grid be achieved. Hence, idr_ref was set to be zero in this simulation study as well. The reference optimum power $\left(P_{Opt\_ref}\right)$ and, the control reference value for the electromagnetic torque ($T_{em\_ref})$ were computed based on: the estimated value of wind speed $\left(V_{est}=10\;m/s\right)$, specified parameters of the 2 MW DFIG system [including gearbox turn ratio (G)=100, optimum power coefficient $(C_{Opt\_max}$) =0.44, torque coefficient $\left(C_{t}=0.061\right)$, optimum tip speed ratio ${lambda}_{Opt}=7.2)$], and by applying Eq. (1), and Eq. (8) respectively. Accordingly, $P_{Opt\_ref}≅1.49\;MW$, and $T_{em\_ref}\approx 8712.1\;N.m$ (the simulation was run based on the principle of generator operation). By employing the values of $P_{Opt\_ref}$ and $T_{em\_ref}$, the reference value for rotor angular speed $\left(\omega_{r\_ref}\right)$ was estimated based on the mathematical relationship between the three parameters, i.e. $\omega_{r\_ref}\approx 171.3\;rad/s$. In addition, the reference value for the rotor quadrature current $\left(i_{qr\_ref}\right)$ was approximated at 1684 A based on Eq. (14). These all reference values were fed into the simulated model of 2 MW DFIG system to evaluate the parameters tracking performances of PI controller (2DOF) under two different conditions: linear (normal) and nonlinear (faulty), the simulation results for the two conditions are respectively displayed according to Figures 17 and 18. Similarly, Table 2 (linear) and Table 3 (nonlinear) present more detail of signal statistics for rotor speed, electromagnetic torque, and rotor currents.

**Table 2:** Signal statistics of control parameters under linear operation of the DFIG WECS.

Control parameters	Signal statistics					
	Max	Min	Peak to Peak	Mean	Media	RMS
Rotor omega_ref	1.713e+02	1.713e+02	0.000e+00	1.713e+02	1.713e+02	1.713e+02
Rotor omega_m	1.909e+02	1.388e+02	5.208e+01	1.709e+02	1.713e+02	1.709e+02
iqr_ref	2.449e+03	−2.449e+03	4.898e+03	1.636e+03	1.672e+03	1.748e+03
iqr	1.716e+04	−9.655e+03	2.682e+04	1.636e+03	1.672e+03	1.883e+03
Tem_ref	1.273e+04	−1.273e+04	2.546e+04	−8.508e+03	−8.694e+03	9.085e+03
Tem	6.663e+04	−1.273e+05	1.942e+05	−8.622e+03	-8.720e+03	1.010e+04
idr_ref.	0.000e+00	0.000e+00	0.000e+00	0.000e+00	0.000e+00	0.000e+00
idr	1.452e+04	-1.153e+04	2.605e+04	4.180e-04	2.764e-1	5.683e+02

**Table 3:** Signal statistics of control parameters under nonlinear operation of the DFIG WECS.

Control parameters	Signal statistics					
	Max	Min	Peak to Peak	Mean	Media	RMS
Rotor omega_ref	1.713e+02	1.713e+02	0.000e+00	1.713e+02	1.713e+02	1.713e+02
Rotor omega_m	2.808e+02	1.055e+02	1.753e+02	1.727e+02	1.713e+02	1.773e+02
iqr_ref	2.449e+03	−2.449e+03	4.898e+03	1.414e+03	2.449e+03	2.254e+03
iqr	2.214e+04	−1.499e+04	3.713e+04	1.463e+03	1.542e+03	2.903e+03
Tem_ref	1.273e+04	-1.273e+04	2.546e+04	−7.350e+03	−1.273e+04	1.172e+04
Tem	8.250e+04	−1.427e+05	2.251e+05	−8.384e+03	−8.760e+03	1.540e+04
idr_ref	0.000e+00	0.000e+00	0.000e+00	0.000e+00	0.000e+00	0.000e+00
idr	2.600e+04	-1.319e+04	3.919e+04	2.228e-03	-1.032e+01	2.980e+03

**Figure 19:**
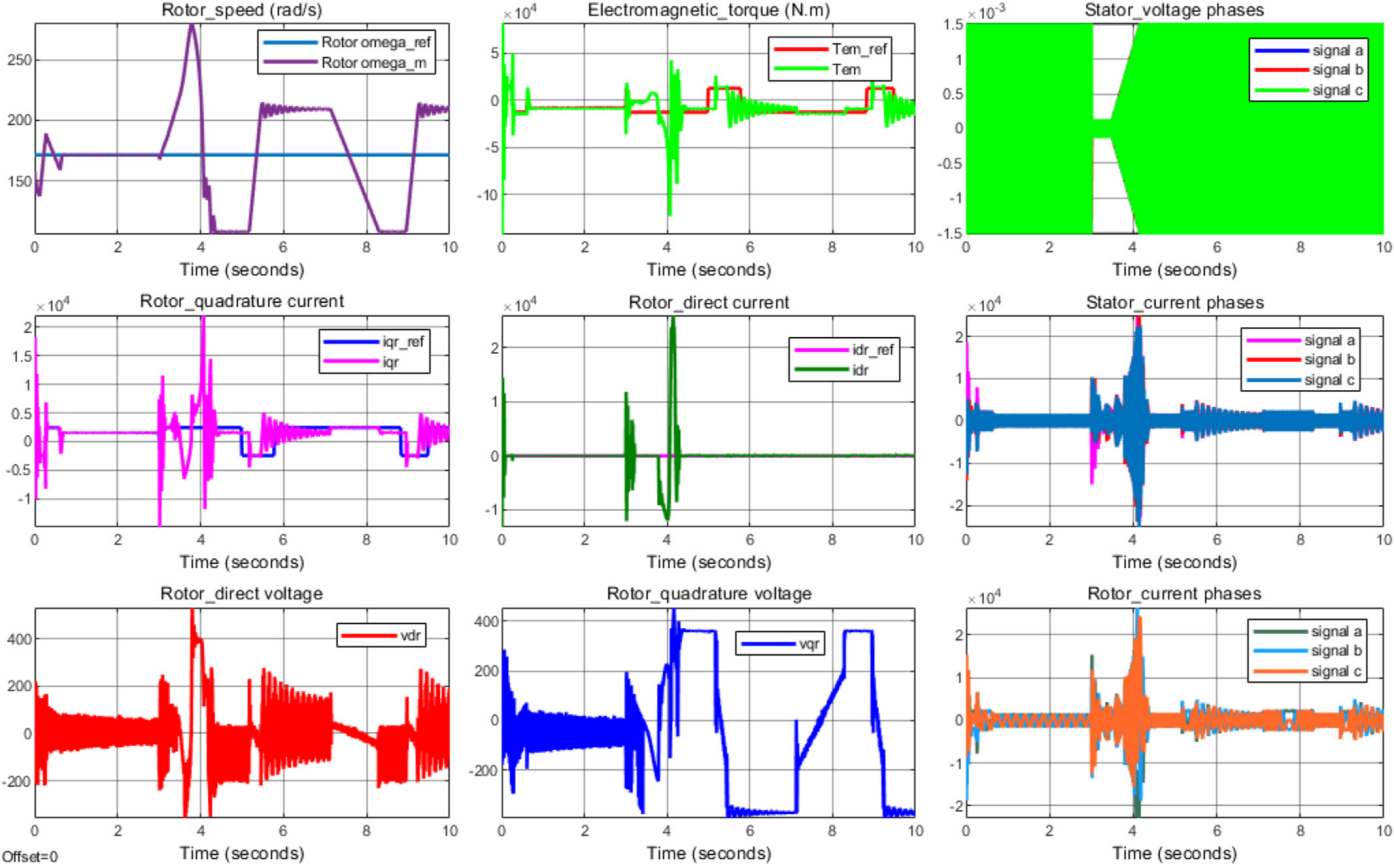
PI (2DOF) performance under system's nonlinear operating condition.

Moreover, Figure 18 displays the simulation results under the condition that when the proposed DFIG system operates by following the exact value of stator voltage, which was specified to be 690 V for this system. On the other hand, Figure 19 demonstrates the simulation results of the same system model under another possible condition that when the specified value of stator voltage would be suddenly dropping, and in this study, the value of the stator voltage was assumed to be dropping by 90 % so that only 0.1∗690V=69V was fed into the developed model. As it can be physically observed, a significant difference in the scale of signal distortion is apparent between the results (rotor speed, electromagnetic torque, direct rotor current, quadrature rotor current, etc.) displayed by Figures 18 and 19. More objective comparison between the two voltage operating conditions can be conducted by analyzing the resulted signal statistics of control parameters presented in Table 2 and Table 3. Indeed, the performance of PI controller (2DOF) under both conditions (normal, and low voltages) can be evaluated based on the respective signal statistics, particularly of rotor speed, electromagnetic torque, and rotor current components, as presented in the two tables.

On the basis of the comprehensive results presented in Table 2, and Table 3, one way of quantifying the performances of PI (2DOF) is made by calculating the total harmonic distortion factor of the rotor alternating current or quadrature current signals, which can be used to determine the level of harmonic distortion generated by the DFIG-based power system. Herein, the total harmonic distortion factor [Eq. (20)] associated with the rotor alternating current is defined as the ratio of the difference between the root mean square (RMS) value of reference quadrature current signal and the RMS value of measured quadrature current signal to the RMS value of reference quadrature current signal expressed in percentage. The resulting total harmonic distortion factor of the rotor quadrature current can be considered to test the robustness of the PI controller (2DOF), and determine the status of produced power quality under both normal, and low voltage operating behaviors of the simulated DFIG system. Hence, the estimated percentage of harmonic distortion factor normally indicate how extreme the peak is in the control signal, and specify the efficiency of the DFIG power system to generate a particular rotor quadrature current. For a modern electrical power system, the percentage distortion factor of a measure alternating (quadrature) current signal is recommended to be not deviating from its reference signal by more than 25% [44]. A higher percentage harmonic factor signals an increasing current distortion level in the power system, which can severely degrade the reliability of power production in addition to threatening the safety of electrical components.

(20) $$I_{qrTHDF}=\frac{I_{qr\_RMS}-I_{qrref\_RMS}}{I_{qrref\_RMS}}\times 100\%$$

By using Eq. (20), and the signal statistics presented in Table 2 and Table 3, the harmonic distortion factor of rotor quadrature current ($I_{qr})$ can be estimated under the normal, and low voltage operating behaviors of the DFIG system as to evaluate the robustness the PI controller (2DOF) against the recommended upper limit of percentage harmonic distortion factor. Accordingly, the quadrature current distortion factor (Figure 18, and Table 2) is computed to be 7.72% when the DFIG system would operate with the normal voltage specification while it rises to 28.79% under a condition that the system would be forced to operate with low voltage value (Figure 19, and Table 3). The result of the harmonic distortion factor under the normal (linear) voltage operating behavior, i.e. 7.72% firmly indicates the robustness of the PI controller (2DOF) as it is clearly observed to be significantly below the recommended upper limit (25%). In this respect, the produced electric power as a function of mean rotor speed & electromagnetic torque, i.e. based on Eq. (16), is estimated to be 1.473 MW, which shows a slightly increased power production compared to the baseline (1.4 MW) provided by [45] for the similar system operating with the same wind speed (10 m/s). In addition, the indicated result of quadrature current distortion factor under the system's linear operating behavior does not result in rotor DC $\left(I_{dr}\right)$ offset; where mean $I_{dr}$ is generated to be 4.180e−04A, which is very close to its reference value (0A). However, the result of the rotor alternating (quadrature) current distortion factor in the case of the system's nonlinear operating behavior has slightly crossed the recommended upper limit, as it has been already indicated; and the electric power of 1.44 MW can be similarly estimated to be produced under this condition as well. The mean rotor DC $\left(I_{dr}\right)$ [Table 3] is also observed to be more deviating from its reference compared to the result for linear operating condition [Table 2].

**Figure 20:**
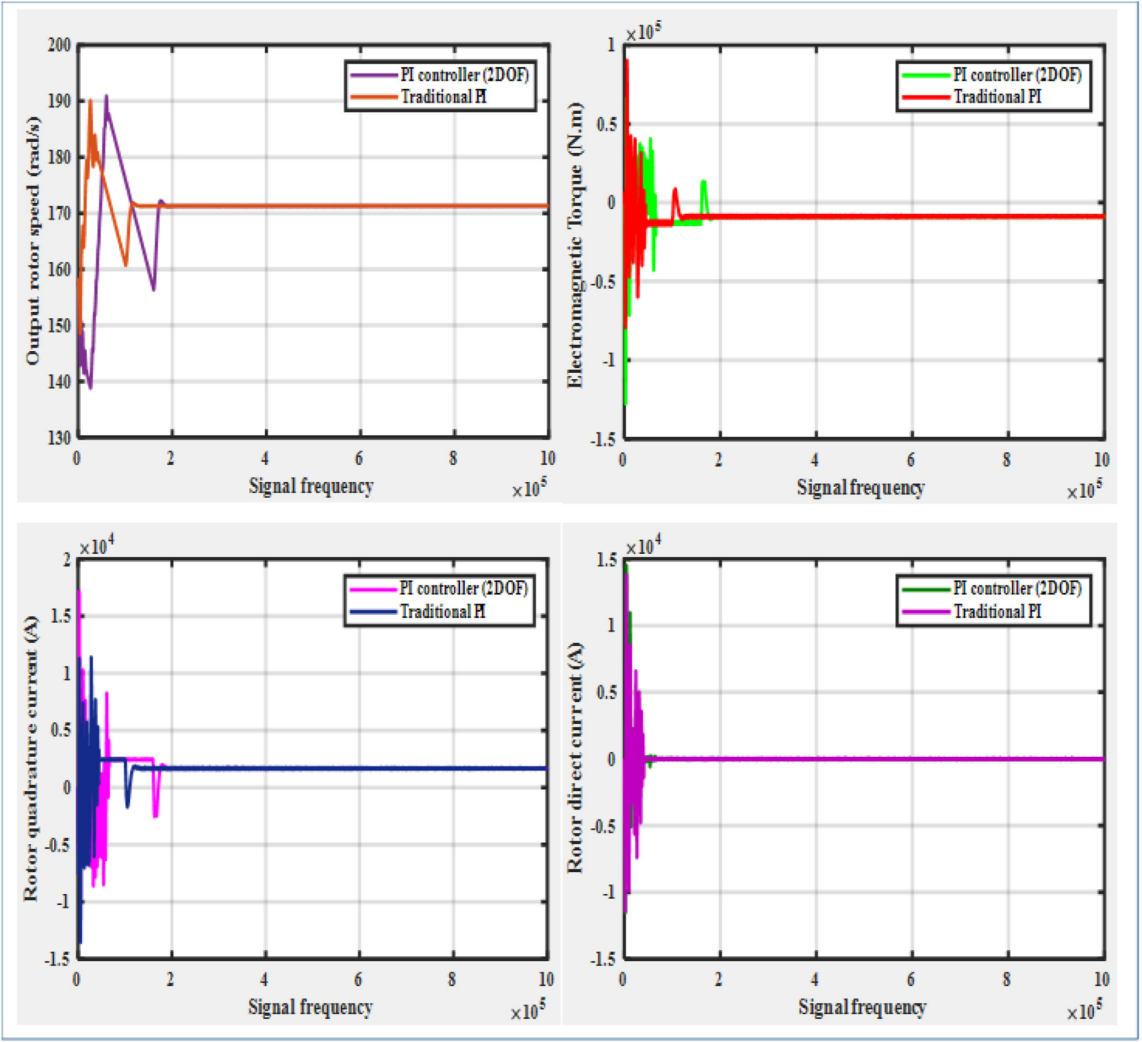
PI controller (2DOF) Vs. Traditional PI controller under linear operation system.

**Figure 21:**
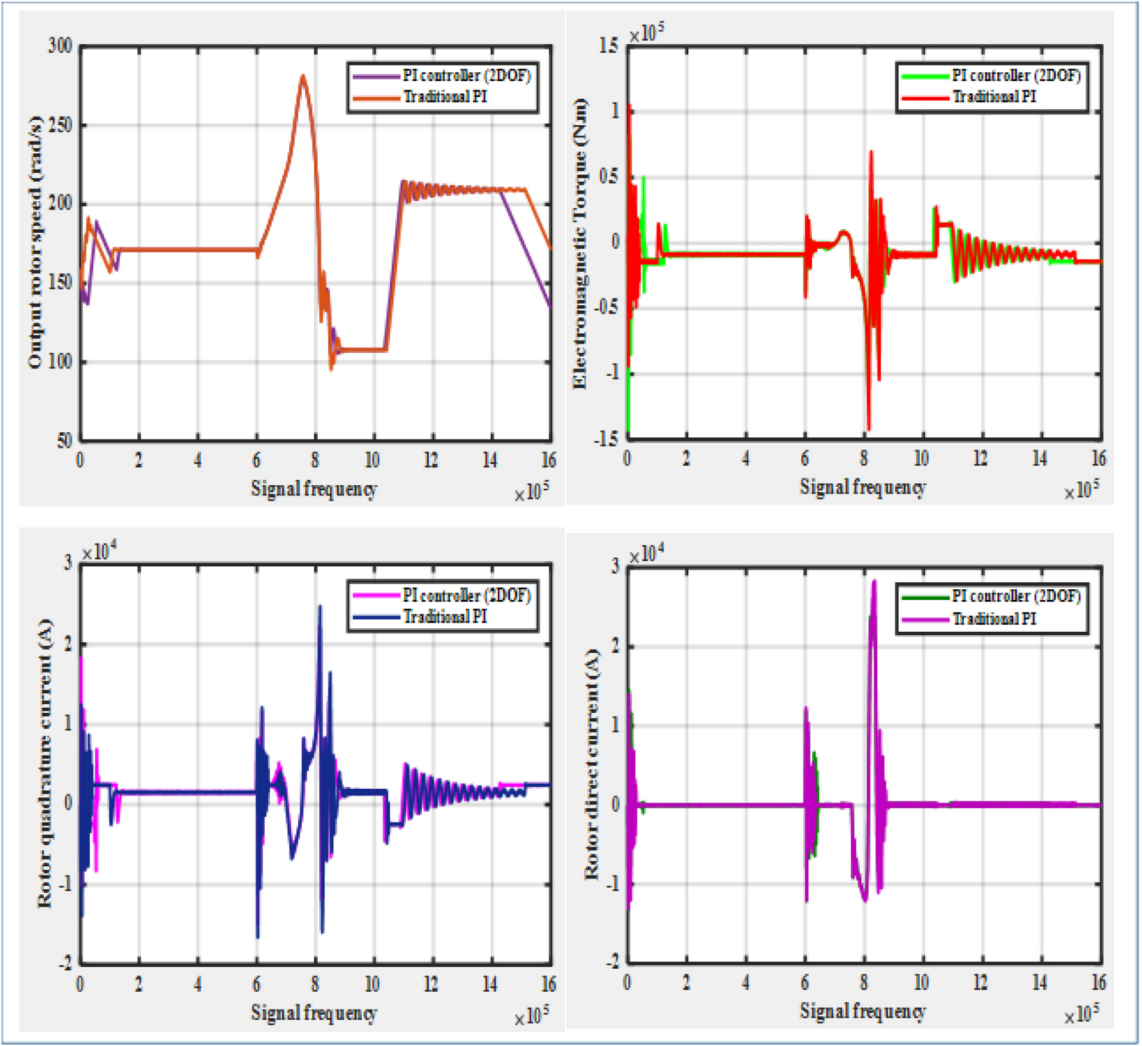
PI controller (2DOF) Vs. Traditional PI controller under nonlinear operation system.

It can be generalized from the previous discussion that the PI controller (2DOF) performs robustly under the DFIG system's linear operating behavior, whereas its performance under nonlinear operation behavior is slightly degrading. Yet, the overall performance of the PI controller (2DOF) can also be additionally evaluated against the performance of the traditional PI controller according the comparisons demonstrated by Figure 20, Figure 21, and Table 4. For instance, as it is shown by Figure 20 for the DFIG system's linear operating behavior, the capability of the PI controller (2DOF) in regulating the rotor quadrature current distortion is better compared with that of the traditional PI controller as their resulting distortion factors are respectively estimated to be 7.72%, and 9.15%. This better capability of PI controller (2DOF) allows the DFIG system to operate over a wider range of rotor speed by ensuring a better performance of the rotor DC reference tracking. Additional comparison of the PI controller (2DOF) performance against the traditional PI controller can be demonstrated under the DFIG system's nonlinear (low voltage) operating condition as illustrated by Figure 21. Under this condition, a significant difference between the PI controller (2DOF), and the traditional PI one has been observed in terms of their capabilities of regulating the rotor current components. Even though the proposed PI controller (2DOF) does not slightly appear to perform as the recommended standard in rejecting the rotor quadrature current distortion, it does not noticeably result in creating rotor DC offset; whereas the much larger rotor quadrature current distortion is generated to create a significant rotor DC offset with the traditional PI controller. Unlike the traditional PI controller that clearly proves to perform poorly under the low voltage operating condition, the new controller allows the DFIG system to spin at a desirable rotor speed, and to produce enough electromagnetic torque in ensuring a reliable power production.

**Table 4:** Proposed PI (enhanced) controller model Vs. Traditional PI controller model.

Controller models	Operating conditions	iqr Harmonic distortion factor (%)	Mean produced electric power (MW)	Mean idr(A)	Comment
PI (2DOF) [Proposed model]– Uses the control gains & set-point weights	Linear	7.72	1.473	4.180e−04	Exhibits a robust overall performance under the system's linear operation; and a moderate performance under the system's nonlinear operation
	Nonlinear	28.79	1.44	2.228e−03	
PI (Conventional) – Based on traditional tuning method	Linear	9.15	1.477	5.826e-−04	Performs well under the linear operating system; and generally does not meet the power quality requirements under the nonlinear operating system
	Nonlinear	35.46	1.42	−6.302e−03	

Further comparison between the performances of the proposed controller, and the traditional controller is summarized in Table 4. The differences between the performances of the two controller models are not appreciably noticeable in their application for the linear operating system as no remarkable gap is observed among their resulting alternating current harmonic distortion levels (7.72% Vs. 9.15%), mean produced electricity (1.473MW Vs. 1.477MW), and mean rotor DC values (4.180e-04A Vs. 5.826e-04A). This generally indicates that both controller models satisfy wind power conversion requirements under the DFIG system's linear operation. On the other hand, under the power system's nonlinear operation, the proposed controller model has proven to demonstrate good performance in handling the power conversion process while the traditional PI controller does not fulfill the required standard for power production. Unlike the traditional PI controller whose performance produces a larger rotor alternating current distortion & fails to keep the rotor DC in the same direction as under the linear operating behavior; a rise in the rotor alternating current distortion level only causes to increase the mean rotor DC value without resulting in a deflection of its direction when the proposed controller model [PI (2DOF)] is employed, which allows to maintain more wind power production by minimizing the possible damages to the DFIG system components that may result from the rotor overcurrent. Hence, the PI controller (2DOF) performs better over the extended range of the DFIG system's operation compared to the traditional PI controller.

## 5 Conclusion and future direction

⁃ This study has tried to examine the robustness of PI controller (2DOF) for application over the broad operating characteristics of DFIG-based wind energy harvesting system by presenting objective and quantitative findings in demonstrating the qualities of its performance. The design models for this controller model, and for different components of the proposed DFIG WECS including mechanical system, aerodynamic system, electrical system, and control system are simulated in MATLAB-SIMULINK so as to conduct the performance evaluations for the simulated PI controller (2DOF) in reference to the overall system design model based on the consideration of two separate conditions, namely: the DFIG WECS's linear operating behavior, and nonlinear operating behavior. The linear operating behavior was modelled to be demonstrated as when the proposed DFIG system would produce wind electric power according to the full voltage specification that was provided by its manufacturer; whereas the nonlinear operating behavior was considered to be a condition under which the DFIG machine's voltage specification would suddenly be falling, which is the most challenging problem in wind power industry nowadays. As part of making the wind power industry more reasonably competitive particularly in terms of its investment costs and wind energy harvesting capability, the DFIG WECS should regularly continue to supply electric power without being potentially impacted by the unfavorable conditions that could arise from different inherent characteristics of wind energy. Hence, the impacts on the wind energy harvesting capability of DFIG-based WECS can be softened by establishing the robust power control system based on the implementation of various control strategies along with the application of different controller models.
⁃ More specifically, this study has developed a PI controller (2DOF) design model as a component of power control system model under a 2 MW power rating-based DFIG WECS simulation in order to assess the controller's performance over extended operation range of the power system. As it can be clearly evident from the findings of this simulated work, the proposed PI controller (2DOF) has generally displayed a robust performance in enabling to maintain a desirable power quality under the system's linear operating characteristics; and it has been observed to show a moderate performance in tracking of the reference rotor current components, rotor speed, and electromagnetic torque under the system's nonlinear operating behavior on the other hand. As it has been indicated in the discussion section of this study, this proposed controller model has been tested to exhibit outstanding overall performance in comparison to the traditional PI controller model; however, more works need to be done for a further minimization of the rotor current harmonic distortion level particularly under the system's low voltage operating behavior commonly known as voltage dip.
⁃ As the DFIG-based WECSs are largely and almost regularly characterized by their nonlinearity in the process of wind energy converting operations, other controller design strategies that can fully circumvent the challenges (including voltage dips) imposed by the nonlinear operating characteristics of these systems should be developed and practically implemented. In response to these challenges, several advanced controller design strategies were quite recently introduced in literatures. These strategies are mainly based on fusing of two different controller designs in aiming to utilize the cumulative of their unique advantages while alleviating their respective limitations in ensuring the wind energy capture over the broader ranges. In this regard, various controller models can be fused with PI controller to enhance its performances under the system's nonlinear operating behavior. For instance, these fusions include: fuzzy logic-PI – where fuzzy logic controller is used to tune PI control gains under grid voltage disturbances in order to maintain the maximum transfer of power; genetic algorithm-PI – where the genetic algorithm can be implemented to tune the performance of PI controller for maintaining an excellent reliability of wind power production under sudden grid voltage variations; and so forth. Yet, further works are still required to be done in the future for more practical confirmation of the effectiveness of these controller design strategies against the real-world wind power industry.

## Declarations

### Author contribution statement

Belachew Desalegn; Desta Gebeyehu; Bimrew Tamrat: Conceived and designed the experiments; Performed the experiments; Analyzed and interpreted the data; Contributed reagents, materials, analysis tools or data; Wrote the paper.

### Funding statement

Belachew Desalegn was supported by Bahir Dar University Institute of Technology R01 BDU – 21 and Wolaita Sodo University PhDR02 WSU – 21-22.

### Data availability statement

Data included in article/supp. material/referenced in article.

### Declaration of interest's statement

The authors declare no conflict of interest.

### Additional information

No additional information is available for this paper.

## Figures and Tables

**Table 5:** Data specification for parameters the proposed DFIG WECS.

Stator frequency	50Hz
Rated stator power	2MW
Rated rotational speed	1500rev/min
Rated stator voltage	690V
Rated stator current	1760A
Rated electromagnetic torque	12732N−m
Pole pair	2
Stator-Rotor turns ratio	1/3
Rated rotor voltage	2070V
Maximum slip	1/3
Rated rotor voltage in reference to stator	230V
Stator resistance	$2.6\times 10^{-3}\;\Omega$
Rotor/Stator leakage inductance	$0.087\times 10^{-3}\;H$
Magnetizing inductance	$2.5\times 10^{-3}\;H$
Rotor resistance in reference to stator	$2.9\times 10^{-3}\;\Omega$
Stator inductance	0.0026H
Rotor inductance	0.0026H
DC bus voltage in reference to stator	325.2691V
